# Alzheimer’s Disease as Type 3 Diabetes: Understanding the Link and Implications

**DOI:** 10.3390/ijms252211955

**Published:** 2024-11-07

**Authors:** Mateusz Kciuk, Weronika Kruczkowska, Julia Gałęziewska, Katarzyna Wanke, Żaneta Kałuzińska-Kołat, Marta Aleksandrowicz, Renata Kontek

**Affiliations:** 1 Department of Molecular Biotechnology and Genetics, Faculty of Biology and Environmental Protection, University of Lodz, Banacha Street 12/16, 90-237 Lodz, Poland; katarzyna.wanke@edu.uni.lodz.pl (K.W.); renata.kontek@biol.uni.lodz.pl (R.K.); 2 Department of Functional Genomics, Medical University of Lodz, 90-752 Lodz, Poland; weronika.kruczkowska@stud.umed.lodz.pl (W.K.); julia.galeziewska@stud.umed.lodz.pl (J.G.); zaneta.kaluzinska@umed.lodz.pl (Ż.K.-K.); 3 Department of Biomedicine and Experimental Surgery, Medical University of Lodz, 90-136 Lodz, Poland; 4 Laboratory of Preclinical Research and Environmental Agents, Mossakowski Medical Research Institute, Polish Academy of Sciences, 02-106 Warsaw, Poland; maleksandrowicz@imdik.pan.pl

**Keywords:** Alzheimer’s disease, type 2 diabetes, glucose metabolism, neurodegeneration, public health

## Abstract

Alzheimer’s disease (AD) and type 2 diabetes mellitus (T2DM) are two prevalent conditions that present considerable public health issue in aging populations worldwide. Recent research has proposed a novel conceptualization of AD as “type 3 diabetes”, highlighting the critical roles of insulin resistance and impaired glucose metabolism in the pathogenesis of the disease. This article examines the implications of this association, exploring potential new avenues for treatment and preventive strategies for AD. Key evidence linking diabetes to AD emphasizes critical metabolic processes that contribute to neurodegeneration, including inflammation, oxidative stress, and alterations in insulin signaling pathways. By framing AD within this metabolic context, we can enhance our understanding of its etiology, which in turn may influence early diagnosis, treatment plans, and preventive measures. Understanding AD as a manifestation of diabetes opens up the possibility of employing novel therapeutic strategies that incorporate lifestyle modifications and the use of antidiabetic medications to mitigate cognitive decline. This integrated approach has the potential to improve patient outcomes and deepen our comprehension of the intricate relationship between neurodegenerative diseases and metabolic disorders.

## 1. Introduction

Alzheimer’s disease (AD) is the predominant neurodegenerative condition in the geriatric population. According to Alzheimer’s Disease International, the incidence of dementia is projected to reach 150 million worldwide by 2050, with AD constituting the majority of cases [1,2]. The etiology of the disease is multifactorial, encompassing genetic predisposition, advanced age, and neuroinflammation. AD is characterized by the early onset of aberrant tau hyperphosphorylation and amyloid-beta (Aβ) plaque formation that ultimately leads to neuronal dysfunction. Consequently, there is a progressive loss of hippocampal neurons and impaired synaptic transmission due to neurotransmitter dysregulation [3]. AD can also result from the genetic impairment of presenilin 1 (*PSEN1*), mainly variants rs63750306 and rs63751235, the presenilin 2 (*PSEN2*) rs63750215 variant, the apolipoprotein E epsilon 4 (*APOE-ε4*) allele, and/or the amyloid precursor protein (*APP*) rs63751263, rs63750264, and rs63750671 variants [4]. Additionally, other factors, including gut microbiota composition, may influence AD development [5]. The diagnosis usually involves brain imaging methods, including computed tomography (CT), positron emission tomography (PET), or magnetic resonance imaging (MRI), and biomarker analyses, including the detection of Aβ plagues, phosphorylated tau proteins, and others in the blood or cerebrospinal fluid [6,7]. Although there is currently no treatment for AD, a variety of pharmaceuticals can enhance patients’ overall quality of life and reduce some of their symptoms. While there may be occasional exceptions, most patients can benefit from FDA-approved acetylcholinesterase inhibitors (AChEIs), such as donepezil, rivastigmine, galantamine, and N-methyl-D-aspartate (NMDA) receptor antagonists like memantine, or newer drugs like lecanemab, donanemab, or aducanumab [8,9].

Type 2 diabetes mellitus (T2DM), also known as adult-onset diabetes, is one of the most common metabolic diseases in the 21st century. About 537 million people worldwide are suffering from the disease, and the number will increase to 643 million by 2030 and 783 million by 2045 [10,11]. The main causes of T2DM are obesity, sedentary lifestyle, poor dietary habits, and insulin resistance [12].

In recent years, there has been growing interest within the scientific community regarding the possible connection between AD and type T2DM, leading some researchers to propose the conceptual term “type 3 diabetes” as a way to describe this link. This term is intended to underscore the potential role of insulin resistance in the brain and its association with neurodegenerative processes in AD. However, it is essential to note that “type 3 diabetes” is not an officially recognized medical or diagnostic category; it has not been adopted by major health organizations such as the World Health Organization (WHO) or the American Diabetes Association (ADA), nor is it used in clinical coding. Rather, “type 3 diabetes” remains a theoretical concept, used by some researchers to highlight the overlap in the pathological mechanisms underlying AD and T2DM. This topic has attracted interest because some studies indicated that the risk of developing dementia, specifically AD, in patients diagnosed with diabetes may be increased. A meta-analysis of 144 prospective studies found a 1.25- to 1.91-fold increased risk of cognitive disorders (cognitive impairment and dementia) [13]. A recent meta-analysis published in 2024 found a 59% increased risk of dementia in diabetic patients compared to non-diabetics [14]. Additionally, Reinke et al. investigated impact of the T2DM duration on the risk of dementia. They found a U-shaped relationship between dementia risk and the duration of type 2 diabetes, with an initial decrease in risk followed by a later increase, underscoring the need for ongoing cognitive monitoring in diabetic patients, particularly several years after diagnosis [15]. A study performed by Yen et al. found that individuals with T2DM followed by hypertension have significantly increased risks of all-cause and vascular dementia, while those with hypertension followed by T2DM also show heightened risks for all-cause, vascular, and other types of dementia, emphasizing the compounded impact of these comorbidities on dementia risk [16]. Nevertheless, several studies do not indicate significant associations. For example, a 5-year longitudinal study found that diabetes is linked to an increased risk of vascular cognitive impairment and vascular dementia but not to AD, suggesting that diabetes primarily impacts dementia through vascular pathways rather than directly influencing AD risk [17]. Kadohara et al. found no overall association between diabetes and the risk of early-onset Alzheimer’s disease (AD), although a weak association was identified in male patients, suggesting that diabetes may not be a significant risk factor for early-onset AD (EOAD) except possibly among men [18], while Hassing et al. found that T2DM is associated with more than twice the risk of developing vascular dementia but is not linked to an increased risk of AD, suggesting a subtype-specific relationship between diabetes and dementia [19].

The common risk factors for the appearance of both AD and T2DM include persistent oxidative stress, obesity, and hypertension. Mechanisms behind the potential link between those two diseases include impaired glucose metabolism and enzymatic pathways, insulin resistance, and elevated inflammation. Consequently, AD is characterized as a metabolic disorder that potentially triggers brain insulin resistance, leading to a cascade of harmful effects including neuronal damage, neurotoxin/Aβ deposition, and neurodegenerative processes [20]. To safeguard against this illness, it is recommended to integrate physical activity and adequate nutrition into the daily routine, utilize probiotic supplements, and abstain from stimulants. Although the area of therapeutic approaches for AD as “type 3 diabetes” is still developing, treatments using common diabetes medications, insulin administration, and targeted therapeutic strategies exist [21,22]. The presented work will concentrate on comprehending the processes, connections, and shared components of T2DM and AD, as well as any potential shared risk factors and possible diagnostic and pharmaceutics approaches.

## 2. Alzheimer’s Disease: Description, Types, and Prevalence in the World

AD, often referred to as Alzheimer’s dementia, is the most prevalent neurodegenerative disorder affecting the elderly. It is characterized by a progressive decline in cognitive functions, including memory loss, diminished cognitive abilities, impaired reasoning, and behavioral changes, ultimately leading to dementia [3]. The first reported case of AD involved a 51-year-old female patient who experienced rapid memory loss and subsequently died at the age of 55 shortly after her diagnosis. This case was documented by German psychiatrist and neuropathologist Alois Alzheimer in 1906, during which he identified the presence of so-called “miliary foci” [23]. Nowadays, AD is recognized as the most prevalent kind of dementia, representing 60–70% of all cases [24].

AD primarily affects individuals over the age of 65. However, it presents in several forms distinguished by the age of onset: early-onset Alzheimer’s disease (EOAD), also called young-onset Alzheimer’s disease (YOAD), and late-onset Alzheimer’s disease (LOAD) [25]. EOAD generally affects individuals between the ages of 45 and 64, accounting for approximately 5–6% of AD cases [26]. Risk factors associated with EOAD include head trauma, cardiovascular conditions, chronic schizophrenia, genetic predispositions, Down syndrome, and lifestyle factors (e.g., poor diet, smoking, alcohol use, and sedentary behavior) [27].

EOAD can be further categorized based on genetic inheritance patterns. Mendelian early-onset AD (mEOAD) is relatively rare and occurs due to specific genetic mutations in genes such as *APP*, *PSEN1*, and *PSEN2*. This form of EOAD has a distinct hereditary pattern and usually presents before age 65. In contrast, non-mendelian early-onset AD (nmEOAD) does not follow a clear genetic inheritance and is thought to result from a complex interaction between environmental and genetic factors. This form of EOAD is more common than mEOAD, with a greater variability in age of onset and disease progression. In general, patients with EOAD tend to have a longer survival time after diagnosis compared to those with LOAD [28].

LOAD is the most prevalent form of AD, representing around 94–95% of cases and typically manifesting after age 65 [25]. Its prevalence increases significantly with each additional five years of age. Key risk factors for LOAD include vascular disease, sleep disturbances (such as insomnia and reduced deep sleep), and genetic factors, especially the *APOE ε4* gene variant, along with the general effects of brain aging [25,29,30].

AD can also be classified into sporadic and familial forms, with familial Alzheimer’s disease (FAD) representing the hereditary subtype. FAD generally appears in individuals between ages 30 and 60, accounting for no more than 1% of all AD cases and 10–15% of early-onset cases [31]. While EOAD encompasses various early-onset forms, FAD is a specific subtype within this category, characterized by its hereditary nature and commonly associated with mutations in *PSEN1*, *PSEN2*, and *APP*. Unlike sporadic EOAD, which can occur without a family history, FAD cases are exclusively inherited and thus fall within the broader category of EOAD [32].

AD is one of the most prevalent types of dementia globally. As of 2023, the WHO and the World Alzheimer Report estimate that over 55 million individuals are affected by dementia, with more than 10 million new cases being identified each year. Projections indicate that this number could rise to 78 million by 2030 and reach 139 million by 2050, driven by factors such as an aging demography, sedentary lifestyles, and various environmental influences. AD constitutes approximately 70% of all dementia cases, making it the most common form of dementia worldwide. As of 2024, countries with the highest prevalence rates of AD include Finland, the United Kingdom, Slovakia, and Japan. Notably, several low- and middle-income countries, particularly in Western Europe, have also reported significant prevalence rates of AD. These findings highlight the growing burden of AD across diverse economic settings, underscoring the need for tailored public health strategies to address the rising prevalence of the disease in both developed and developing regions (for reference see https://www.who.int/news-room/fact-sheets/detail/dementia, https://www.alzint.org/about/dementia-facts-figures/dementia-statistics/ (accessed on 6 November 2024)).

## 3. Pathogenesis of AD

### 3.1. Tau Protein Hyperphosphorylation

The tau protein is encoded by the microtubule-associated protein tau (*MAPT*) gene located on chromosome 17q21. This protein consists of three major structural domains: the N-terminal projection domain, the proline-rich region (PRR), and the microtubule-binding domain (MTBD), along with a C-terminal domain. The primary biological functions of the MAPT protein include the stabilization of microtubules, particularly within axons, facilitating axonal transport and providing structural support for postsynaptic scaffolding [33,34]. In the human brain, tau exists in six major isoforms, and its functional properties are influenced by the specific isoform present as well as its phosphorylation status. In the context of AD, the isoforms featuring either three repeats or four repeats in their microtubule-binding domains are particularly significant [35,36]. While phosphorylation is a natural post-translational modification of the tau protein, abnormal hyperphosphorylation can lead to significant alterations in its structure and function. This pathological hyperphosphorylation is often associated with increased activity of proline-directed protein kinases (PDPKs), which can disrupt the normal interactions and stability of tau, contributing to neurodegenerative processes [37,38]. The hyperphosphorylation of tau protein, especially on Ser262, Thr231, and Ser235 residues [39], leads to its excessive aggregation inside neurons due to the conformation, charge changes, and a buildup of NFTs [40]. The disruption of axonal transport is a well-established consequence of tau dysfunction, which also significantly impairs synaptic plasticity. During hyperphosphorylation, tau undergoes abnormal self-assembly, forming paired helical filaments (PHFs) and straight filaments (SFs). These aggregated tau species are neurotoxic, contributing to tauopathies by interfering with neuronal function and accelerating neurodegeneration. In AD, the accumulation of NFTs is markedly increased, with studies demonstrating up to a four-fold rise compared to healthy individuals. This pronounced aggregation of tau underscores its central role in AD pathogenesis and progression [38].

### 3.2. Amyloid Beta (Aβ) Deposition

Aβ is a soluble protein biologically involved in synaptic plasticity, memory processes associated with the hippocampus, neuroprotection, and cellular metabolism and survival [41]. It is encoded on chromosome 21 by the *APP* gene mostly expressed in synapses. The synthesis of Aβ occurs through a multistep process. It begins in the endoplasmic reticulum, where APP is synthesized and then transported to the Golgi apparatus for maturation, after which it is directed to the plasma membrane. From this point, APP can be processed via two distinct pathways: the amyloidogenic and non-amyloidogenic pathways [41,42,43]. The amyloidogenic pathway involves the beta-site amyloid precursor protein cleaving enzyme 1 (BACE1) cleavage of APP, leading to the production of Aβ peptides that aggregate to form amyloid plaques. In contrast, the non-amyloidogenic pathway is initiated by α-secretase cleavage within the Aβ region, which precludes Aβ formation and instead generates the neuroprotective sAPPα fragment [44].

In the amyloidogenic pathway, γ-secretase cleaves the β-carboxy-terminal fragment (β-CTF) to produce Aβ peptides, primarily Aβ_1-40_ and Aβ_1-42_. In the non-amyloidogenic pathway, cleavage by γ-secretase generates the p3 peptide, corresponding to amyloid-β peptides Aβ_17-40_ and Aβ_17-42_. Following cleavage, APP can either be recycled back to the Golgi apparatus or transported via endosomes. Aβ is subsequently recycled or degraded by lysosomes. The excessive production of Aβ, which is insoluble at high concentrations, can be triggered by genetic factors, aging, overproduction through the amyloidogenic pathway, impaired clearance, or comorbidities such as diabetes [45,46]. The accumulation of Aβ peptides results in the generation of plaques, which in turn disrupt Aβ function, resulting in synaptic damage, oxidative stress, and, most notably in AD, progressive neuronal loss and memory decline [45,47]. Among the two major forms of Aβ peptides—Aβ_1-40_ and Aβ_1-42_—Aβ_1-42_ is thought to play a more prominent role in AD due to its higher aggregation propensity, even though Aβ₄₀ is more abundant in the human brain [48].

### 3.3. Influence of Gut Microbiota

A relatively new hypothesis for the occurrence and formation of AD is its connection with the structure of the intestinal microbiota. It garnered great attention in 2012 and is still being studied today [49]. The intestinal microbiota consists of more than 1000 strains of bacteria, as well as yeast, viruses, protozoa, parasites, and archaea [50,51]. The gut–brain axis plays a key role in the process of connecting microglia in the brain with the intestinal microbiota in the digestive system [52]. This interconnection is primarily based on biochemical signaling [53]. The first link occurs within the nervous system, where the vagus nerve, part of the central nervous system (CNS), establishes a crucial connection between the brain and the intestine, which itself contains a nervous system known as the enteric nervous system. Additionally, neurotransmitters such as γ-aminobutyric acid (GABA), dopamine, serotonin, and glutamate are produced by both the brain and the gut. The immune system plays a significant role in this relationship as well; the gut–brain axis also controls immune responses. These responses can be influenced by factors such as stress and obesity, which will be discussed further in this article [54,55,56]. The validity of these findings has been supported by research conducted on germ-free animals [57,58,59]. Alterations in gut health, particularly dysbiosis—characterized by changes in bacterial metabolites and the composition of intestinal strains—have been correlated with various diseases, including Parkinson’s disease, depression, diabetes, multiple types of cancer, and AD [60,61,62]. A notable change in the microbiome of individuals with AD is the reduced content of supportive microorganisms, such as *Firmicutes*, which play a crucial role in carbohydrate breakdown and butyrate synthesis [63], *Bifidobacterium*, probiotic bacteria that regulate gut and immune system function [64], and *Eubacterium rectale*, known for its antioxidative properties and involvement in fiber fermentation and butyrate production [65]. Furthermore, an increased presence of certain bacterial strains, including *Proteobacteria*, *Escherichia/Shigella*, and *Porphyromonas gingivalis* (often found in the oral microbiome), is associated with severe inflammatory responses and has been linked to tau protein and Aβ deposition in the brain [66,67]. These shifts in the microbiome also involve the release of neuroactive compounds into the surrounding environment. Both Gram-negative bacteria, including *Escherichia* and *Proteobacteria*, and Gram-positive bacteria, such as *Firmicutes,* are the integral components of the gut microbiota. Their production of short-chain fatty acids (SCFAs), trimethylamine (TMA) (and its oxidized form, trimethylamine N-oxide [TMAO]), as well as lipopolysaccharides has been linked to immune responses and the modulation of brain activity associated with AD pathology [68,69,70]. It is important to note that individuals with a family history of AD and exposure to risk factors should consider dietary approaches such as the dietary approaches to stop hypertension (DASH), Mediterranean-DASH intervention for neurodegenerative delay (MIND), and Mediterranean diets (MeDS), along with the inclusion of prebiotics and probiotics and regular physical activity [58,71,72].

### 3.4. Gene Mutations

As previously noted, genetic factors and their mutations play a crucial role in the pathology of AD. To date, over 40 genes have been associated with this condition; however, only about 10 genes have been confirmed as contributors to the disease’s onset and risk [73,74]. AD is influenced by a complex interplay of genetic factors rather than a single genetic cause. The first gene linked to AD was the *APP* gene, identified by Goate et al. in 1991 [75]. As described in previous sections, APP is a transmembrane protein involved in the synthesis and deposition of Aβ. The amyloid precursor protein family of proteins, including amyloid precursor-like proteins 1 and 2 (APLP1 and APLP2), has been the subject of intensive study due to its involvement in neurodegenerative diseases. Among these, APLP1 is particularly noteworthy. APLP1 is expressed in neurons and, when mutated, serves as a significant genetic component defining the features of AD [76]. Pathogenic mutations in the *APP* gene (32 mutations identified) predominantly occur in exons 16 and 17 and are frequently point mutations, deletions, or duplications. Numerous studies have confirmed that these mutations lead to an increased imbalance between Aβ_40_ and Aβ_42_, consequently impairing clearance and resulting in the abnormal deposition of Aβ plaques [77,78,79].

The presenilin genes, *PSEN1* and *PSEN2*, are undeniably related to the etiology of AD, with approximately 340 pathological mutations identified in AD patients, predominantly missense mutations [80,81]. *PSEN1* mutations can manifest with various symptoms, including seizures, while *PSEN2* mutations, which are less common, are associated with specific types of dementia, such as Lewy body dementia and frontotemporal dementia [82,83]. Similarly to *APP* mutations, alterations in *PSEN1* and *PSEN2* are also implicated in the increased production of Aβ42, its aggregation, and the phosphorylation of tau protein [84,85].

The most frequently confirmed gene associated with AD is *APOE*, located on chromosome 19. Its primary function is to facilitate cholesterol transport [86]. The ε4 allele of the *APOE* gene is strongly associated with an increased risk of AD. Specifically, individuals carrying one copy of this allele have a three-fold increased risk of developing the disease, while those with two copies face a twelve-fold increased risk [87]. However, the *APOE* gene does not solely exert negative effects; the ε2 allele exhibits protective properties, including the suppression of Aβ deposition, the preservation of cognitive function, and a 50% reduction in the likelihood of AD onset [88,89].

*APOE ε4* is implicated in various pathogenic processes in AD that collectively contribute to neuronal degeneration. These mechanisms encompass dysregulated Aβ metabolism, synaptic dysfunction, neuroinflammation—hallmarks of AD—and altered cholesterol homeostasis, which may also be linked to diabetes [90,91,92]. Furthermore, genome-wide association studies (GWASs) have identified numerous other genes potentially associated with AD, including disintegrin and metalloproteinase domain-containing protein 10 (*ADAM10*), ATP-binding cassette sub-family A member 7 (*ABCA7*), bridging Integrator 1 (*BIN1*), cortactin-CD2-associated protein (*CD2AP*), ephrin type-A receptor 1 (*EPHA1*), phosphatidylinositol binding clathrin assembly protein (*PICALM*), sortilin-related receptor 1 (*SORL1*), complement receptor type 1 (*CR1*), clusterin (*CLU*), microtubule-associated protein tau (*MAPT*), and others. However, further genetic research is required to elucidate the roles that these genes play in the onset of AD [4].

### 3.5. Neuroinflammation

Inflammation represents a crucial area for further investigation into the pathogenesis of AD. Numerous investigations have shown that while acute inflammation can initially serve a protective role, chronic inflammation in the brain is a significant contributor to the development and progression of AD.

Recent research by Jin et al. has highlighted the critical role of astrocyte dysfunction in AD pathology. Astrocytes in the brains of AD patients exhibit notable morphological and functional differences compared to those in unaffected individuals. In AD, astrocytes display heightened reactivity, leading to the increased release of pro-inflammatory cytokines, such as interleukin 1 beta (IL-1β) and TNF-α. Additionally, these dysfunctional astrocytes are less effective in clearing excess glutamate from the synaptic cleft, which contributes to excitotoxicity and subsequent neuronal damage. Furthermore, the diminished expression of adhesion proteins, such as claudin-5, compromises the functionality of the BBB, resulting in heightened permeability and potential neuroinflammatory responses [93].

Addressing these pathological alterations in astrocyte function presents a promising therapeutic target for slowing AD progression, particularly in its early stages when neuronal damage may still be reversible. However, it is essential to recognize that astrocytes also possess numerous neuroprotective and regenerative functions within the brain. Thus, a major challenge in developing novel therapeutic strategies lies in selectively modulating astrocytic function to enhance their protective roles without adversely affecting their normal physiological activities.

## 4. The Link Between Alzheimer’s Disease and Diabetes

The relationship between AD and diabetes, particularly T2DM, has been the subject of extensive research over many years. T2DM, the most prevalent form of diabetes, is often associated with obesity and a sedentary lifestyle. When not properly managed, elevated blood sugar levels can lead to damage to various organs, including the brain. Recent studies have suggested a significant connection between T2DM and AD. Both conditions share common pathological features, including insulin resistance, impaired glucose metabolism, and amyloid protein aggregation. Due to these overlapping mechanisms, AD is sometimes referred to as the “diabetes of the brain” or “type 3 diabetes mellitus” [94,95,96]. According to the Alzheimer’s Association and multiple long-term population studies, individuals with T2DM may have an increased susceptibility to AD. Using cross-sectional neuroimaging and cognitive data on a large cohort of human individuals from the UK Biobank, scientists described neurocognitive effects that are separately linked with age and T2DM. Findings revealed that T2DM was linked to cognitive deficits, particularly in executive function and processing speed, along with significant gray matter atrophy in the ventral striatum, cerebellum, and putamen. These structural and functional changes closely resembled the neurodegenerative effects of aging but manifested earlier in T2DM patients, with longer disease duration associated with greater neurodegeneration. Metformin, a common treatment for T2DM, did not trigger improvements in brain function or cognitive abilities in patients. This suggests that T2DM may speed up the aging process in the brain and that managing blood sugar alone might not be enough to protect cognitive health in diabetic patients [97]. Another study involving 5653 participants found that individuals with diabetes experienced a 45% faster decline in general cognitive abilities, memory, and reasoning compared to non-diabetic individuals. In contrast, those with normoglycemia experienced a 29% faster cognitive decline. Additionally, individuals with poorly controlled diabetes exhibited more rapid memory and reasoning deterioration, indicating that both disease duration and glycemic control are crucial factors influencing cognitive decline in middle-aged T2DM patients [98].

### 4.1. Insulin Dysregulation in the Brain

Insulin dysregulation in the brain has garnered heightened interest in recent years due to growing evidence linking impaired insulin signaling to various neurological and cognitive disorders. Insulin receptors are widely distributed across the brain, particularly in regions crucial for memory, learning, and cognitive functions, including the hippocampus, cerebral cortex, and hypothalamus [99]. More than a decade before the onset of AD symptoms, there is a notable decline in brain glucose metabolism [21,100,101]. This decline underscores the association between dementia and abnormal energy metabolism, as insulin signaling plays a vital role in glucose metabolism, neurotransmitter release, and synaptic plasticity—key processes for optimal cognitive performance [102]. Furthermore, insulin dysregulation in the brain may contribute to the accumulation of Aβ plaques and NFTs, the two hallmark pathologies of AD [103]. Impaired insulin signaling in the hypothalamus, a region that regulates energy balance and appetite, may also contribute to metabolic disorders such as T2DM and obesity, both of which elevate the risk of cognitive decline [104,105].

The transport of insulin over the blood–brain barrier (BBB) is a meticulously regulated process vulnerable to disturbance by multiple factors. Conditions such as obesity, inflammation, hyperglycemia, and dyslipidemia can reduce insulin permeability across the BBB [106,107]. Moreover, aging and systemic insulin resistance are associated with a decline in the cerebrospinal fluid (CSF) to plasma insulin ratio and a reduction in insulin receptor expression. The decreased insulin availability and signaling in the brain can have a profound influence on neuronal function and cognitive processes [21,108,109]. At the molecular level, glucose uptake in the brain primarily relies on glucose transporter protein 1 (GLUT1) and glucose transporter protein 3 (GLUT3), which function independently of insulin. However, insulin facilitates glucose uptake via glucose transporter protein 4 (GLUT4), particularly in synaptic regions during periods of heightened activity. This insulin-dependent glucose transport becomes crucial during intense cognitive tasks and synaptic plasticity events [108,110]. Brain insulin resistance can arise from several molecular mechanisms. Increased activity of protein tyrosine phosphatase 1B (PTP1B) dephosphorylates insulin receptor substrate 1 (IRS-1), inhibiting insulin signaling. Elevated levels of pro-inflammatory cytokines, including tumor necrosis factor-alpha (TNF-α), monocyte chemoattractant protein-1 (MCP-1), C-reactive protein (CRP), and various interleukins, suppress GLUT4 expression and disrupt IRS-1 function. Oxidative stress further exacerbates these effects by degrading IRS, inhibiting GLUT4 trafficking, and triggering the accumulation of ceramides and protein aggregates. These processes collectively lead to cytokine activation, mitochondrial dysfunction, and, ultimately, neuronal death [111,112].

Insulin normally plays a protective role by suppressing Aβ toxicity, reducing the formation of Aβ oligomers, and regulating extracellular Aβ degradation through THE modulation of insulin-degrading enzyme (IDE) activity. However, diminished insulin signaling impairs these protective mechanisms, potentially accelerating AD progression [113,114]. The consequences of brain insulin resistance are far-reaching and contribute significantly to cognitive decline and neurodegenerative processes. Impaired glucose metabolism resulting from insulin resistance leads to inadequate glucose levels in the brain, compromising intracellular insulin signaling. This metabolic dysregulation increases the activity of BACE [115,116,117]. Prolonged hyperinsulinemia leads to the downregulation of insulin receptors at the BBB, decreasing insulin transport into the brain. The reduced expression of insulin and insulin-like growth factor (IGF-1/2) receptors correlates with increased glycogen synthase kinase 3 beta (GSK-3β) activity and elevated *APP* mRNA expression [118,119]. Both of these pathologies contribute to Aβ accumulation and NFT development—hallmarks of AD. Moreover, reduced insulin signaling decreases O-GlcNAcylation and promotes tau hyperphosphorylation, further contributing to the formation of NFTs. Additionally, reduced glucose metabolism impairs glutamate uptake by astrocytes, potentially leading to excitotoxicity [120,121]. The decrease in UDP-N-acetylglucosamine (UDP-GlcNAc), a byproduct of glucose metabolism, promotes the hyperphosphorylation of tau and APP, further exacerbating neurotoxicity [115,116,117].

Ball and his colleagues [122] investigated the relationship between certain blood molecules and neuroinflammation. They studied the effects of nine specific molecules (lauric acid, asparagine, fructose, arachidonic acid, aminoadipic acid, sorbitol, retinol, tryptophan, and niacinamide) on mouse neurons, chosen because of their potential connection to AD, T2DM, or both. The study found that neurons reacted differently depending on whether the blood molecules were linked to positive or negative outcomes in AD and diabetes [122]. This suggests that these molecules may modulate brain inflammation by altering neuronal communication. Notably, two inflammatory mediators, MCP-1 and interleukin 9 (IL-9), were produced at varying levels depending on the molecule’s disease association [122]. A further investigation revealed a troubling trend in people with both AD and T2DM: a significant decrease in the phosphorylation of markers related to insulin signaling (insulin receptor beta (IRβ), phosphatidylinositol 3,4,5-trisphosphate (PI3K), 3-phosphoinositide-dependent protein kinase 1 (PDK1), protein kinase B (AKT) and GSK-3β). This implies that in people with both disorders, the intracellular communication necessary for insulin activity was more impaired than for people with only one disease [123]. Additionally, a meta-analysis of cohort studies indicated that individuals with diabetes have a higher likelihood of developing AD compared to the general population, a trend particularly pronounced in Eastern populations [124]. The intricate relationship between impaired insulin signaling, glucose metabolism, neuroinflammation, and the accumulation of neurotoxic proteins such as Aβ and hyperphosphorylated tau underscores the critical role of insulin in maintaining brain health and highlights the potential of targeting insulin-related pathways for the prevention and treatment of neurodegenerative diseases.

### 4.2. Accumulation of Amyloid Beta (Aβ)

Aβ deposition is one of the key hallmarks of AD. Although Aβ protein is normally present in synapses within the CNS, pathological circumstances lead to its increased deposition in regions like the frontal cortex and hippocampus [125]. This phenomenon is largely driven by the impaired function of the *APP* gene, which results in the excessive production and deposition of Aβ in the brain [126]. However, this is not the only factor contributing to Aβ deposition—insulin resistance plays a critical role in this pathological process. Insulin resistance, which can be induced by conditions such as obesity, hypertension, inflammation, and T2DM, is closely linked to Aβ pathology [12]. The connection is rooted in the synthesis pathway of Aβ. As previously discussed, there are two main pathways for Aβ generation: the non-amyloidogenic pathway, which is influenced by insulin, and the amyloidogenic pathway, which also undergoes minor modifications due to insulin and is strongly associated with AD and T2DM [127]. Importantly, only the amyloidogenic pathway contributes to plaque deposition, while a properly functioning non-amyloidogenic pathway is responsible for the clearance of Aβ [45,128].

The correlation between the *APP* gene and insulin in the brain is centered on the modulation of *APP* expression and the clearance of its product—Aβ [129]. Insulin plays a key role in the non-amyloidogenic pathway of APP, which is not associated with amyloid plaque deposition. However, the dysregulation of insulin function in this pathway significantly contributes to Aβ deposition [130,131]. The balance between the amyloidogenic and non-amyloidogenic pathways is regulated by the PI3K/AKT/GSK-3β signaling pathway. This pathway enables individuals without insulin resistance to maintain normal Aβ clearance. In this process, PI3K is activated in response to the binding of insulin or insulin-like growth factor-1 (IGF-1) to their respective receptors. These receptors phosphorylate insulin receptor substrate (IRS) proteins. The phosphorylated IRS proteins then bind to and activate PI3K. Once activated, PI3K phosphorylates the membrane lipid phosphatidylinositol 4,5-bisphosphate (PIP2), converting it into phosphatidylinositol 3,4,5-trisphosphate (PIP3). PIP3 serves as a docking site for AKT and its upstream activator PDK1 at the cell membrane. AKT is then phosphorylated and activated by PDK1 and other kinases, which initiates a range of downstream signaling pathways [22,118,131,132]. Active AKT also phosphorylates GSK-3β on specific resides threonine 308 (Thr308) and serine 473 (Ser473). This phosphorylation inactivates GSK-3β [131], promoting the non-amyloidogenic pathway, in which α-secretase cleaves *APP*, releasing soluble APP alpha (sAPPα), a neuroprotective form of APP that supports Aβ clearance [118,133]. Insulin resistance disrupts the PI3K/AKT/GSK-3β signaling pathway. This leads to increased GSK-3β activity due to reduced phosphorylation, decreased AKT activity caused by unphosphorylated IRS, and lower IGF-1 levels. These changes impair signal transduction, promoting the amyloidogenic processing of APP and subsequent Aβ accumulation [121,130]. Given that insulin resistance and these pathway disruptions are closely linked to type T2DM, numerous studies indicate a higher risk of AD in diabetic patients [134,135,136]. Additionally, Aβ aggregates can impair cognitive function when deposited in non-neural tissues such as the liver, skin, intestines, and heart [137,138,139].

In a study conducted by Wijesekara et al. [140], the relationship between Aβ deposition and insulin resistance was examined using a double-transgenic (dTg) mouse model. Insulin and glucose were injected intraperitoneally into the animals, and their blood glucose levels were monitored with a conventional glucometer. The results demonstrated that induced insulin resistance led to signs of β-cell impairment, hyperglycemia, AD symptoms, and potential Aβ plaque formation in the dTg model [140]. Another connection between T2DM and AD involves protein misfolding, particularly islet amyloid polypeptide. This misfolding increases the likelihood of Aβ aggregation in the brain. Chronic inflammation, a shared characteristic of both diseases, also contributes to cognitive impairment and amyloid deposition [141,142]. Medical interventions that address insulin resistance, such as drugs commonly prescribed for T2DM, including liraglutide, dulaglutide, and lixisenatide, have had encouraging effects in alleviating AD symptoms. For example, liraglutide reduced Aβ_1-42_ levels, lowered overall plaque deposition, mitigated the effects of insulin resistance, and enhanced cognitive function and memory in the mice models of AD [143,144]. Lixisenatide also positively impacted neuroinflammation, reduced amyloid deposition, and decreased the presence of NFTs, while dulaglutide aided in the restoration of cognitive abilities in mice and AD patients [145,146,147]. Additionally, insulin administered through intranasal or intravenous routes has been shown to improve cognitive function and inhibit plaque deposition and formation in AD patients [148,149,150].

### 4.3. Accumulation of Tau Protein

After hyperphosphorylation, tau protein misfolds and aggregates into NFTs in the neuronal cytoplasm. Tau protein aggregates are not broken down by autophagy, causing their accumulation. This buildup eventually causes oxidative stress within the cell, and the mitochondria’s production of ROS triggers apoptotic signals, intensifying neuronal death. Neuronal degeneration leads to the loss of synaptic connections, which are critical for memory retention since mature neurons cannot regenerate [151,152,153]. Normally, the protein phosphatase 2 A (PP2A) phosphatase regulates and prevents tau hyperphosphorylation and misfolding. However, changes in glucose metabolism have been shown to lead to abnormal tau hyperphosphorylation, as hypothermia can inhibit PP2A activity in the brain. People with diabetes often experience hypothermia. This may be linked to a decrease in PP2A enzyme activity, which can cause the excessive phosphorylation of the tau protein and increase the risk of dementia and AD [154,155,156]. As previously noted, insulin dysfunction leads to tau hyperphosphorylation, but it also affects the mitogen-activated protein kinase (MAPK) and AKT signaling pathways, both of which are crucial for tau regulation [132]. In neurons containing hyperphosphorylated tau, insulin accumulates and forms oligomers, a characteristic observed in AD and other common tauopathies [118,135,157].

It is also important to recognize that tau protein is expressed in various regions of the body, not just the brain [158,159]. Research by Balczon et al. [160] demonstrated that infection-induced (*P. aeruginosa*) endothelial amyloids impair memory by inhibiting synaptic plasticity, while Lin et al. [161] proposed that nosocomial pneumonia could be a source of neurotoxic amyloids. Furthermore, Scott et al. [162] provided evidence of reduced dendritic spine density in brain neurons in pneumonia mice models. In this context, the presence of tau in the pancreas seems particularly significant. In pancreatic islet β-cells, tau plays a regulatory role in glucose-stimulated insulin secretion. Interestingly, elevated tau levels in T2DM patients paradoxically enhance insulin secretion, potentially contributing to hyperinsulinemia. However, the chronic overexpression of tau may disrupt β-cell function. Conversely, tau suppression has been shown to normalize glycemic control by promoting insulin secretion and inhibiting microtubule assembly. These findings indicate that tau protein could be a potential therapeutic target for managing both neurodegenerative and metabolic disorders [163].

A 2021 observational study involving 1350 patients found that T2DM can influence neurodegeneration along the AD continuum. Among the participants, those with diabetes exhibited higher total tau levels compared to non-diabetic patients. Additionally, a significant association was observed between diabetes and CSF tau levels in *APOE E4+* carriers, but not in *APOE E4*- individuals. This study underscored the importance of diabetes in AD pathophysiology and its implications for treatment [164]. Another study, published in 2024, focused on a diabetic AD mouse model to investigate changes in tau phosphorylation patterns in the brain. The results revealed a unique tau phosphorylation signature in diabetic AD mice, identifying the potential tau-related kinases and signaling pathways involved in the interaction between diabetes and AD [165]. A meta-analysis by Lu et al. [166] found that individuals with both diabetes and cognitive impairment experienced a more rapid decline in Aβ_1-42_ levels and an increase in tau compared to those without diabetes. They also discovered that the association between phosphorylated tau levels and diabetes was stronger than the link with prediabetes.

**Figure 1:**
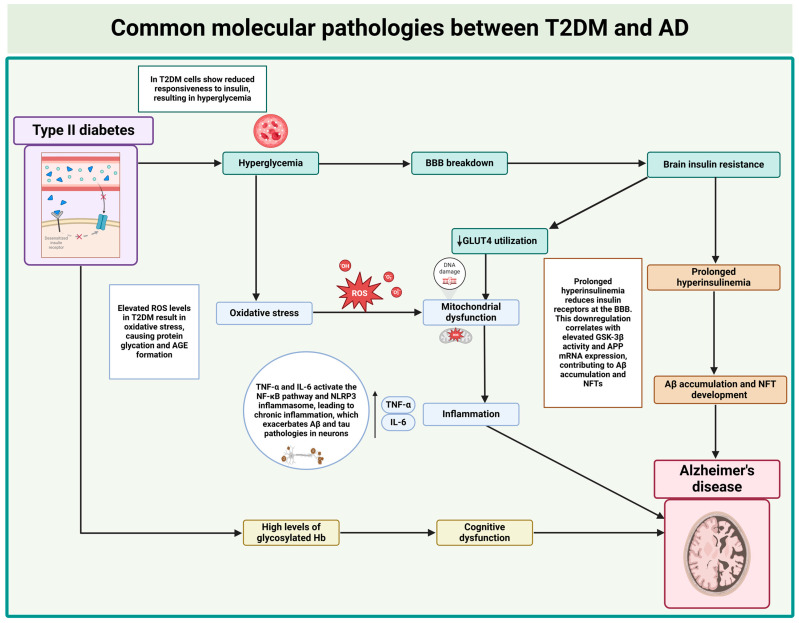
Common molecular pathologies between type 2 diabetes mellitus (T2DM) and Alzheimer’s disease (AD). In T2DM, cells become less responsive to insulin, leading to hyperglycemia. Hyperglycemia results in oxidative stress and inflammation, both of which contribute to mitochondrial dysfunction, reducing glucose metabolism and accelerating AD pathology. The BBB breakdown limits insulin transport into the brain, causing brain insulin resistance, which prolongs hyperinsulinemia. This downregulation of insulin receptors correlates with increased glycogen synthase kinase 3 beta (GSK-3β) activity and elevated amyloid precursor protein (APP) mRNA expression, resulting in the accumulation of Aβ and neurofibrillary tangles (NFT). High levels of pro-inflammatory cytokines, such as tumor necrosis factor α (TNF-α) and interleukin-6 (IL-6), activate the nuclear factor kappa-light-chain-enhancer of the activated B cells (NF-κB) pathway and the NOD-like receptor pyrin domain-containing 3 (NLRP3) inflammasome, perpetuating chronic inflammation, which exacerbates amyloid beta (Aβ) and tau pathologies in neurons. These inflammatory processes are further sustained by the oxidative stress and mitochondrial dysfunction present in both T2DM and AD. High levels of glycosylated hemoglobin (Hb), another consequence of hyperglycemia, are linked to cognitive dysfunction. Created with BioRender.com, accessed on 25 October 2024.

This evidence indicates an intricate network of common molecular pathologies between T2DM and AD, as summarized in Figure 1.

## 5. Common Risk Factors

**Figure 2:**
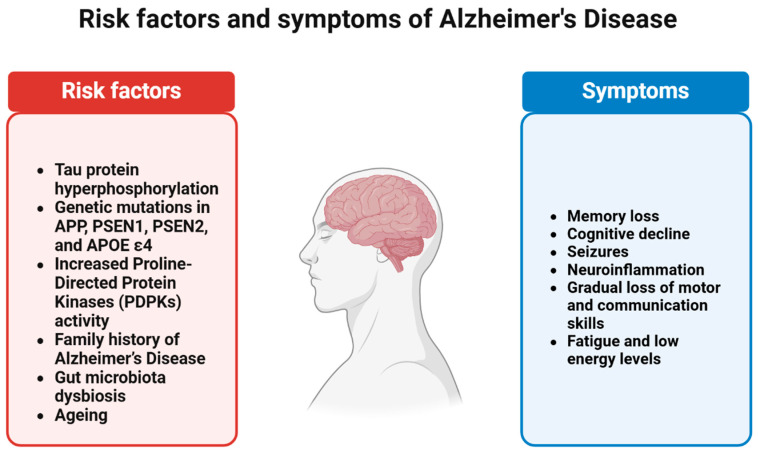
The risk factors and symptoms associated with Alzheimer’s Disease. Abbreviations: APOE ε4—apolipoprotein E4; APP—amyloid precursor protein; PDPKs—phosphoinositide-dependent protein kinases; PSEN1—presenilin 1; PSEN2—presenilin 2. Created with BioRender.com, accessed on 25 October 2024.

The connection between T2DM and AD is largely driven by overlapping risk factors that are common for both conditions. Key factors, such as insulin resistance and elevated blood sugar levels, play a crucial role. As individuals age, the likelihood of developing either disease increases. According to the Alzheimer’s Association, obesity is another shared risk factor, as is chronic low-grade inflammation, which is frequently triggered by a high-fat diet. Additionally, poor cardiovascular health—including high blood pressure and elevated cholesterol levels—is a significant concern for those at risk of either disease. Genetics and oxidative stress also appear to contribute, with certain genes impacting both conditions [95,167]. This section highlights original research papers that have identified these common risk factors, emphasizing the genetic aspects and role of oxidative stress, both of which are frequently encountered in modern times. Figure 2 provides a summary of the risk factors and symptoms associated with AD.

### 5.1. Genetic Aspects

AD etiology is complex with no definitive causation. Recently, a method to pinpoint AD patients who may be more susceptible to T2DM has been developed. The researchers applied a non-negative matrix factorization (NMF) approach to extract differentially expressed gene (DEG) subgroups, identifying 241 candidate genes (such as actin beta (*ACTB*), serine incorporator 3 (*SERINC3*), and zinc finger MIZ-type containing 1 (*ZMIZ1*)) that share common features with AD and T2DM, such as dysregulated T cell selection and chemokine pathways. This approach could facilitate the development of personalized treatments for AD [168]. Another study similarly utilized NMF on DEGs from human cortical neurons to uncover shared genetic patterns between AD and T2DM. Through protein–protein interaction (PPI) network analysis, the researchers identified key biomarkers and therapeutic targets. The most significant shared genes were cyclin-dependent kinase inhibitor 1A (*CDKN1A*), collagen type XXII alpha 1 chain (*COL22A1*), eukaryotic initiation factor 4A (*EIF4A*), glial fibrillary acidic protein (*GFAP*), solute carrier family 1 member 1 (*SLC1A1*), and vimentin (*VIM*) [169]. Additionally, Wang et al. [170] analyzed two large genome-wide association studies (GWASs) to identify the genetic loci associated with both T2DM and AD. By using conditional false discovery rate (cFDR) analysis, they identified 78 single nucleotide polymorphisms (SNPs) linked to AD, 58 of which were unique to Europeans. Among the significant genes identified—tumor protein p53-inducible nuclear protein 1 (*TP53INP1*), the translocase of outer mitochondrial membrane 40 (*TOMM40*), and reduced nicotinamide adenine dinucleotide ubiquinone oxidoreductase complex assembly factor 6 (*NDUFAF6*)—were those involved in mitochondrial dysfunction and oxidative stress, both of which are implicated in the development of AD and T2DM [170]. These findings offer new insights into the shared genetic basis of T2DM and AD, providing potential avenues for novel treatment strategies.

### 5.2. Oxidative Stress

Oxidative stress plays a critical role in the pathophysiology of insulin resistance and T2DM, two conditions that elevate the risk of AD. In T2DM, hyperinsulinemia can impede Aβ breakdown and promote glucose metabolism, leading to an increase in reduced nicotinamide adenine dinucleotide (NADH) and flavin adenine dinucleotide (FADH2) levels and ATP production. This excess adenosine triphosphate (ATP) generates more ROS, which in turn causes the non-enzymatic glycosylation of proteins, lipoproteins, and nucleic acids, forming advanced glycation end products (AGEs). Receptors for AGEs (RAGEs) are upregulated in AD mouse models and have been linked to AD pathogenesis [171,172]. Oxidative stress is also known to reduce glucose metabolism. As the brain consumes the most energy in the body, primarily through glucose utilization, oxidative stress may impair glucose metabolism by damaging the enzymes involved in glycolysis, the tricarboxylic acid cycle, and ATP biosynthesis [100,173].

Liu et al. [174] employed metabolomics to investigate metabolic changes in the serum and pancreas of human amyloid precursor protein and presenilin 1 (*APP/PS1*) double-transgenic mice AD model. Their study found that AD triggers global glucose metabolism dysfunction, including oxidative stress, decreased energy metabolism, and hyperglycemia. Additionally, a redox proteomics study identified oxidatively modified brain proteins (such as ATP synthase, aldolase, and α-enolase) in AD, MCI, and Aβ animal models. These proteins disrupt key processes, including energy metabolism, protein degradation, synaptic function, and neurotransmission. Disruptions align with AD hallmarks such as synapse loss, amyloid plaque deposition, and NFTs formation, suggesting that oxidative stress may drive AD pathogenesis [175].

Moreover, T2DM can exacerbate neurodegenerative processes, leading to brain atrophy, reduced glucose metabolism, and CNS insulin resistance. This, in turn, promotes early Aβ accumulation, which can further impair CNS insulin signaling, initiating a cycle of brain injury, inflammation, and oxidative stress. This creates a feedback loop where oxidative stress and insulin resistance reinforce each other, driving the progression of both T2DM and AD [153,176]. Understanding the shared role of oxidative stress in T2DM and AD could open the door to new therapeutic strategies targeting this common pathogenic mechanism, though further research is needed to fully unravel the complex interactions between oxidative stress, T2D, and AD.

### 5.3. Obesity

Obesity significantly increases the likelihood of developing T2DM and AD. Individuals with obesity are six times more likely to develop T2DM compared to those with average weight, and this risk escalates with the severity of obesity. Specifically, individuals with a body mass index (BMI) over 40 kg/m^2^ face an even higher risk of developing diabetes. Obesity contributes to insulin resistance, a critical factor in the onset of T2DM, as excess fat tissue diminishes the cells’ responsiveness to insulin [177]. A case–cohort study conducted in 2020 demonstrated that maintaining normal body weight is essential for reducing the risk of T2DM. Obesity (BMI ≥ 30 kg/m^2^) emerged as the strongest risk factor, regardless of genetic predisposition or lifestyle habits. Even among individuals with a low genetic risk and healthy lifestyle, those who were obese had over an eight-fold increased risk of developing T2DM compared to the average-weight individuals in the same risk group [178]. Obesity may elevate the risk of AD through various mechanisms, including chronic inflammation, impaired brain insulin signaling, vascular damage, and hormonal and neurotransmission changes (e.g., leptin and glutamate). These factors contribute to neurodegeneration, Aβ plaque deposition, and other hallmarks of AD [179,180]. A 2023 observational cohort study explored the relationship between brain atrophy and protein accumulation (Aβ and tau) in both obese individuals and those with AD. The study found that brain atrophy patterns in obese individuals closely resembled those observed in AD, suggesting that weight management could potentially benefit brain health in older adults by slowing cognitive decline and reducing the risk of developing AD [181]. Conversely, Cho et al. [182] examined the relationship between BMI and AD risk in the Asian population. The researchers discovered that underweight individuals have a heightened likelihood of developing AD compared to those who maintain an average weight. Conversely, individuals who are overweight or obese appear to have a reduced risk of AD. Importantly, within the underweight group, the risk of AD increased in proportion to the underweight severity. These results underscore the importance of maintaining a healthy weight throughout life to mitigate the risk of AD. Public health initiatives aimed at actively screening underweight individuals for AD and promoting healthy weight management could be beneficial in alleviating the burden of AD [182]. More confusingly, obesity in mid-life is a recognized risk factor for AD dementia, but obesity in late-life has been suggested as a protective condition. Late-life obesity may appear as protective against AD-related brain atrophy due to the confounding effects of weight loss, which itself is associated with brain atrophy and predates cognitive decline, thus supporting the hypothesis of reverse causation in the obesity paradox for AD risk [183]. This highlights the need for further research to clarify these relationships.

### 5.4. Hypertension

Hypertension is another common risk factor for both T2DM and AD, though much of the research focuses primarily on the relationship between hypertension and AD. Interestingly, T2DM increases the likelihood of developing high blood pressure, suggesting a potential link between overall metabolic health and brain function [184,185,186]. High blood pressure disrupts insulin signaling in two key ways: it damages blood vessels, making insulin delivery more challenging, and, through chronic inflammation, it further weakens the cells’ response to insulin [187]. A recent study from 2021 found that controlling blood pressure could be helpful for people with type 2 diabetes who are already taking medication to manage their blood sugar levels and have not experienced any memory problems. This might lower their chances of dying or having heart problems [188]. Hypertension may also elevate the risk of cognitive impairment through several mechanisms. It damages the brain’s blood vessels, restricting the delivery of oxygen and nutrients to brain cells, which can lead to strokes and hemorrhages. Furthermore, high blood pressure disrupts communication between different regions of the brain and triggers inflammation that may exacerbate AD [189,190]. A study found that middle-aged individuals with moderately elevated systolic blood pressure (stage 1 or 2 hypertension) have an 18% to 25% higher risk of developing AD later in life compared to those with normal blood pressure [191]. Other researchers explored whether hypertension directly impacts AD or if it acts indirectly. They discovered that high blood pressure may indirectly raise the risk of AD by affecting the brain’s blood vessels, particularly through the narrowing of arteries in the circle of Willis. This narrowing likely limits blood flow to the brain and impairs its ability to remove waste products. Both a reduced blood flow and the buildup of waste could contribute to AD. The study suggests that high blood pressure’s effect on the circle of Willis may be a crucial factor in explaining its role in increasing AD risk [192]. Gabin et al. [193] examined the relationship between blood pressure and AD risk. Among individuals over 60, a lower systolic blood pressure was associated with a reduced risk of AD. However, for those under 60, the relationship was more complex. A higher systolic blood pressure and pulse pressure were linked to a greater risk of AD, but only in individuals who reported taking blood pressure medications [193]. Lee et al. aimed to address the inconsistent findings regarding the impact of blood pressure control on the risk of dementia, particularly focusing on how different blood pressure levels might affect various dementia subtypes (AD and vascular dementia). The authors sought to clarify the relationship between systolic and diastolic blood pressure and dementia risk, considering factors like antihypertensive treatment and the presence of comorbidities. The study suggests that managing blood pressure may have complex implications for dementia risk, particularly with respect to lower blood pressure in older adults who are on antihypertensive treatment. The U-shaped association indicates that both excessively high and low blood pressure could contribute to increased dementia risk, suggesting that careful management is critical, especially in those with comorbid conditions. This underscores the need for personalized treatment strategies in hypertension management to balance the potential risks and benefits regarding cognitive health in older adults. Further research is warranted to explore these associations and their clinical significance in the context of dementia prevention strategies [194]. In 2024, Lennon et al., motivated by previous randomized controlled trials and longitudinal studies suggesting that continued antihypertensive medication in older adults may lower the risk of all-cause dementia, investigated specific effects on Alzheimer’s dementia versus non-Alzheimer’s dementia. The research aimed to determine whether a history of hypertension or antihypertensive treatment affects the risks of Alzheimer’s dementia and non-Alzheimer’s dementia in late life and to identify the optimal blood pressure levels for reducing these risks. The study underscored the importance of managing hypertension in late life as a strategy to mitigate the risk of Alzheimer’s dementia, indicating that antihypertensive treatment is beneficial. While treated hypertension correlates with a lower risk of Alzheimer’s dementia, both treated and untreated hypertension present increased risks for non-Alzheimer’s dementia. Additionally, the findings highlight that while a single measurement of blood pressure did not correlate with Alzheimer’s dementia risk, diastolic blood pressure may have a complex U-shaped relationship with non-Alzheimer’s dementia risk, suggesting that monitoring and managing blood pressure over time is crucial for cognitive health in older adults. This research supports the ongoing efforts to develop targeted interventions for hypertension management to protect against dementia, particularly Alzheimer’s dementia, in the aging population [195].

### 5.5. Poor Diet

Maintaining a balanced diet is essential for managing glucose levels, particularly for individuals with diabetes. It is crucial to avoid both overeating and excessively strict diets. Monitoring daily food intake can improve blood glucose and insulin concentrations and can potentially slow neurodegeneration [196,197]. One study examined how different blood sugar management strategies (intensive vs. less aggressive) affected elderly patients with both T2DM and AD. Chen et al. [198] concluded that, for these patients, a less aggressive approach to blood sugar control—allowing for slightly higher target blood sugar levels—might be more beneficial [198].

A balanced intake of protein, fat, and carbohydrates, alongside low-glycemic index (GI) foods, is also critical for managing glucose levels [199]. A 2018 meta-analysis revealed that the Mediterranean diet is one of the most effective diets for improving glycemic control in T2DM patients. It also improves cardiometabolic health and lowers mortality in T2DM patients [200,201]. A study by Mattei and colleagues found that following the Mediterranean diet was linked to better cognitive abilities and blood sugar control. People who followed the Mediterranean diet more closely also had better memory after two years [202]. This diet emphasizes fruits, vegetables, legumes, whole grains, seafood, moderate dairy products (e.g., yogurt and reduced-fat cheese), wine, and olive oil for cooking while limiting red meat intake [203].

Another plant-based diet, the DASH, focuses on reducing processed foods and saturated fats. A hybrid of the MeDS and DASH diets, known as the MIND, has been designed to promote neuroprotection. Morris et al. demonstrated that adherence to the MIND diet was linked to a lower incidence of AD [204]. However, while the DASH and MIND diets show promise, they have not been studied as extensively as the Mediterranean diet, and the evidence supporting their effectiveness is not as robust [205].

The ketogenic diet (KD) has also gained attention. Characterized by low carbohydrate intake (around 5%), high fat intake (around 80%), and moderate protein intake (15%), KD has been linked to positive outcomes in AD patients [206,207]. Medium-chain triglyceride (MCT) oil, a key component of the KD, may support mitochondrial function and enhance ketogenesis in AD patients [208,209]. Additionally, the KD may improve lipid profiles, promote weight loss, and lower HbA1c levels [210,211].

A growing body of preclinical and clinical research supports the benefits of KD for AD, with improvements in mitochondrial function, optimized gut microbiota composition, decreased neuroinflammation, and reduced oxidative stress among the potential mechanisms [212,213,214,215,216].

Finally, supplements may play a role in managing both T2D and AD, although their effects can vary between individuals. Some evidence suggests positive outcomes in patients with T2D and AD. For example, a 2019 study found that *Lactobacillus acidophilus* had an anti-diabetic effect and improved epithelial barrier function [217]. Supplementing with vitamins C and E may also be beneficial for managing diabetes and cognitive decline [218,219,220].

### 5.6. Sedentary Lifestyle

While a healthy diet is a key player in managing T2DM and AD, there are other ways to support your overall health. AD and T2DM are linked to insulin resistance and physical inactivity. Firstly, physical exercise can enhance insulin BBB transport and the binding of insulin to brain endothelial cells in mice [221]. Aerobic exercise training (AET) can be used as a preventive measure or supplementary treatment approach by regulating the expression of certain microRNAs (miRNAs) involved in the disease pathogenesis. Consequently, five miRNAs, namely miR-21, miR-29a/b, miR-103, miR-107, and miR-195, were identified as potential targets for treating AD and T2DM [222]. A cohort study from 2022 examined the link between physical activity (PA) changes and dementia risk in patients with new-onset T2DM. Results showed that regular PA was associated with lower risks of all-cause dementia, AD, and vascular dementia. The study suggested that regular PA should be encouraged to prevent dementia in high-risk populations and those with new-onset T2DM [223]. A pilot randomized controlled trial from 2024 proved that high-intensity low-volume and low-intensity high-volume functional training is beneficial in improving a few biochemical, physical, and mental functioning parameters in elderly T2DM patients experiencing cognitive decline [224]. On the other hand, Shabab et al. investigated the effect of high-intensity interval training (HIIT) and moderate-intensity continuous training (MICT) combined with metformin. They analyzed how it influenced impaired memory in rats with diabetes. The study found that diabetic groups treated with metformin and HIIT and MICT exercises improved latency, staying time, entrance frequency, and oxidative stress caused by diabetes. These exercises also attenuated serum glucose levels, similar to the effects of metformin [225]. Zhong et al. found that regular physical activity benefited people without dementia in several aspects. The intervention increased levels of substances in the spinal fluid that are associated with a healthy brain [226]. An umbrella review of existing meta-analyses looked at exercise and AD. They found strong evidence suggesting that regularly receiving the recommended amount of exercise helps prevent AD. Exercise also seems to improve brain function, physical abilities, and daily living activities for people with AD, although more research is needed to find the best exercise routines for these patients [227]. A randomized controlled trial conducted by Australian researchers between 2004 and 2007 evaluated the effects of a structured 6-month physical activity program on cognitive function in older adults. The study demonstrated cognitive improvements that persisted over an 18-month follow-up period. The proposed mechanisms underlying these benefits include enhanced glucose uptake by skeletal muscle and other peripheral tissues, as well as a reduction in systemic insulin resistance. This suggests that physical activity not only improves overall metabolic health but also exerts neuroprotective effects that may be beneficial in AD management [228].

### 5.7. Alcohol and Tobacco Use

Stimulants, such as smoking and alcohol, play a significant role in everyday life. However, quitting smoking and reducing alcohol consumption is especially important for individuals with T2DM or AD, as these habits have detrimental effects on both heart and brain health [229,230]. Smoking, in particular, increases the risk of developing T2DM and can worsen outcomes for those already diagnosed. It also negatively impacts brain health, raising the risk of AD and other forms of dementia. Smoking has various harmful effects on the brain, including increasing oxidative stress, promoting cerebrovascular disease, triggering heightened inflammatory responses, and potentially directly contributing to the neuropathology of AD [231,232].

A cohort study from 2023 found that cumulative smoking exposure was associated with cognitive decline in older adults without dementia, highlighting the need for further population-based research in order to understand this connection [233]. A meta-analysis of 19 studies revealed that current smokers were more likely to experience cognitive impairment and dementia. Smokers had a higher risk of developing AD, vascular dementia, and any form of dementia compared to nonsmokers. Furthermore, smokers showed more significant declines in mini-mental state examination (MMSE) scores during the follow-up period, indicating a stronger likelihood of dementia and cognitive decline [234].

When it comes to T2DM, one study examined the relationship between smoking and glycemic control in Japanese male patients with T2DM. Results indicated that smoking raised HbA1c levels, with the increase being proportional to the number of cigarettes smoked daily and the duration of smoking (packs per year). However, after quitting smoking, HbA1c levels decreased linearly with the years of cessation. This study demonstrated a dose- and time-dependent relationship between smoking and glycemic control, underlining the importance of smoking cessation in diabetes management [235].

A 2024 study explored the long-term impact of smoking and diet quality as modifiable risk factors for cardiovascular disease (CVD) and all-cause mortality among both current and former smokers. Over an eight-year follow-up, 40 deaths (4.20%) and 94 CVD cases (9.80%) were recorded. Among current smokers, a lower-quality diet was associated with an increased mortality risk. In contrast, light smokers with a healthy diet had a reduced risk of both CVD and all-cause mortality. This protective effect was particularly evident among ex-smokers with high diet quality scores [236].

Alcohol consumption also affects diabetes management by disrupting blood sugar regulation. It can lead to unpredictable fluctuations in glucose levels, impair the liver’s ability to regulate glucose, cause hypoglycemia, reduce insulin effectiveness, increase appetite, and lead to poor dietary choices. Additionally, alcohol consumption can worsen conditions like insulin resistance, contributing to the development or exacerbation of T2D [237,238]. Chronic heavy drinking is also linked to cognitive impairment and an elevated risk of dementia [239,240].

A study by Oba-Yamamoto et al. investigated the effects of alcohol on blood sugar levels in healthy individuals. They found that consuming alcohol along with glucose led to more frequent episodes of hypoglycemia compared to glucose alone, suggesting that alcohol can contribute to reactive hypoglycemia when paired with sugary drinks [241].

In terms of dementia risk, a 2019 study examined how alcohol consumption impacts older adults. It found that moderate drinking (7–14 drinks per week) was associated with a lower risk of dementia in individuals without MCI. However, for those with MCI, alcohol did not show any clear benefits, and heavy drinking was linked to faster cognitive decline. Interestingly, in people without MCI, both abstaining from alcohol and heavy drinking were associated with lower cognitive function compared to moderate drinking. These findings suggest that moderate alcohol consumption may be safe for brain health in older adults without MCI, but caution is necessary for those with MCI [242].

A study by Yen et al. explored how alcohol affects memory and cognitive function in older adults. They found that heavy drinking was associated with a lower risk of cognitive decline in individuals aged 60–69, but the opposite was true for people aged 70 and above, where heavy drinking increased the risk. Additionally, factors such as high blood pressure and lower income were linked to a heightened risk of cognitive decline [243].

Common risk factors for AD and T2DM are summarized in Table 1.

## 6. Treatment

### 6.1. Pharmacological Interventions

Targeting insulin resistance has gained attention as a promising therapeutic approach for AD. One of the drugs under investigation in this area is pioglitazone, a member of the thiazolidinediones (TZDs) class, typically used to treat T2DM. Pioglitazone works by enhancing insulin sensitivity. Its mechanism of action involves the activation of peroxisome proliferator-activated receptor gamma (PPAR-γ), which binds to specific DNA sequences to regulate the expression of genes associated with glucose and lipid metabolism [244].

Pioglitazone was associated with a reduced risk of developing dementia in patients with DM. This effect appeared to be particularly significant in patients with a history of stroke or ischemic heart disease. These findings suggest that pioglitazone’s neuroprotective and anti-inflammatory properties may contribute to cognitive preservation, possibly through improved insulin sensitivity, reduced vascular damage, and the modulation of neuroinflammation [245].

Preclinical studies have demonstrated that pioglitazone can improve synaptic activity, which supports learning and memory functions. However, most clinical trials conducted so far have been too small or too short in duration to observe significant therapeutic effects. As a result, the efficacy of pioglitazone in treating AD remains uncertain, and further research is needed to establish its potential benefits [246,247].

For example, the combination of pioglitazone and insulin therapy in T2DM patients has raised concerns about an elevated risk of developing AD, possibly through mechanisms involving increased adiposity, insulin resistance, or changes in the brain’s inflammatory and metabolic environment [248].

The expanding research into glucose metabolism also offers a promising avenue for understanding the pathophysiology of AD. Hypometabolism, or reduced glucose metabolism in the brain, is one of the hallmark biomarkers of AD, often appearing long before the onset of clinical symptoms. This phenomenon is particularly pronounced in the temporoparietal regions, where it correlates with the cognitive deficits seen in AD patients. Independent of the future development of T2DM, individuals with blood glucose levels near the lower limit of normal (72–99 mg/dL) may be at an elevated risk for developing AD [249]. Thus, glucose dysregulation plays a critical role in the early pathogenesis of AD.

In the context of AD as a potential diabetes-related disorder, metformin, a widely prescribed biguanide for managing type II diabetes, has emerged as a promising therapeutic candidate. Metformin primarily acts by reducing hepatic glucose production and enhancing insulin sensitivity. However, beyond its well-established glucose-lowering effects, metformin has also demonstrated neuroprotective potential in AD. This is attributed to its anti-inflammatory, antioxidant, and neurodegenerative-modifying properties, which may mitigate neuronal damage and support cognitive function. These multifaceted effects suggest that metformin could play a role in addressing metabolic dysfunction and neurodegeneration in AD, making it a candidate for further investigation in the context of AD treatment [250].

One particularly compelling mechanism through which metformin may exert its effects on AD is by modulating autophagy, a critical cellular process responsible for the degradation and clearance of pathological proteins such as Aβ and tau, which are central to AD pathology. Preclinical studies suggest that metformin enhances autophagic activity, thereby facilitating the removal of toxic protein aggregates from the brain. Furthermore, metformin positively influences mitochondrial function through the activation of adenosine monophosphate-activated protein kinase (AMPK), a key regulator of cellular energy homeostasis. By activating AMPK, metformin promotes mitochondrial biogenesis and modulates inflammatory pathways, which may collectively attenuate the progression of AD. These mechanisms highlight metformin’s potential as a therapeutic candidate for addressing both protein aggregation and metabolic dysfunction in AD [251].

In a study conducted by Ou and colleagues, metformin demonstrated neuroprotective effects in *APP*/*PS1* transgenic mice through the modulation of key signaling pathways, including the AMPK/mechanistic target of rapamycin (mTOR)/ribosomal protein S6 kinase (S6K)/BACE1 and AMPK/NFκB. Metformin treatment significantly increased phosphorylated AMPK levels while reducing the expression of phosphorylated mTOR, S6K, P65NFκB, and BACE1, all of which are involved in APP processing and inflammation. Notably, metformin’s activation of AMPK was observed in glial cells, suggesting that it may contribute to the reduction in Aβ accumulation in these cells. The reversal of metformin’s effects by an AMPK inhibitor confirmed that its neuroprotective actions are AMPK-dependent. This finding highlights metformin’s potential therapeutic benefit in AD, particularly by targeting mitochondrial dysfunction, a hallmark of neurodegenerative conditions [252].

Further evidence of metformin’s neuroprotective effects comes from the study by Li et al., which demonstrated that metformin inhibits cyclin-dependent kinase 5 (CDK5) activity and reduces histone H1 phosphorylation in the hippocampus of transgenic mice. The study also reported that metformin treatment led to a significant reduction in phosphorylated tau protein at serine residues 202 and 404, which are critical sites associated with tau pathology in AD. Following 10 days of metformin administration, behavioral testing revealed marked improvements in spatial memory, as well as a reversal of long-term synaptic potentiation (LTP) deficits, highlighting metformin’s potential to restore cognitive function. These findings suggest that metformin may exert therapeutic effects by targeting both tau pathology and synaptic dysfunction in AD [253].

In conclusion, metformin presents a compelling candidate for AD treatment, owing to its multifaceted mechanisms that target key aspects of AD pathology, including autophagy, mitochondrial dysfunction, and neuroinflammation. While the results of preclinical studies are encouraging, further clinical trials are essential to elucidate metformin’s full therapeutic potential in human AD patients, particularly regarding long-term cognitive outcomes and disease progression.

Growing interest has been directed toward the potential use of glucagon-like peptide-1 (GLP-1) receptor agonists in AD, based on their success in treating T2DM [254,255] and their effects on neural health. GLP-1 receptor agonists, such as liraglutide, semaglutide, and exenatide, exert neuroprotective actions by promoting insulin sensitivity, reducing neuroinflammation, and enhancing neuronal survival. These agents activate GLP-1 receptors in the brain, which are widely expressed in regions associated with cognitive function, including the hippocampus and cortex. Animal studies have shown that GLP-1 receptor agonists can reduce Aβ plaques and phosphorylated tau levels, while also improving synaptic function and cognitive outcomes. Furthermore, GLP-1 receptor agonists appear to exert antioxidant effects and improve mitochondrial function, potentially counteracting the cellular energy deficits that contribute to AD progression [256,257,258,259,260,261,262,263,264,265,266]. Several clinical trials are underway to evaluate the cognitive effects of GLP-1 receptor agonists in AD patients, with preliminary data showing promise in slowing cognitive decline in mild to moderate cases of AD [266,267,268,269,270,271].

Similarly, sodium–glucose cotransporter-2 (SGLT-2) inhibitors, another class of antidiabetic agents, are being explored for their neuroprotective potential in AD. SGLT-2 inhibitors, such as empagliflozin [272,273,274] and dapagliflozin [275,276,277,278], work by reducing glucose reabsorption in the kidneys, thus improving glycemic control in T2DM patients. Beyond their role in glucose regulation, these drugs have demonstrated the protective effects against oxidative stress and inflammation, which are critical contributors to AD pathology. SGLT-2 inhibitors are also known to enhance ketone production, which may serve as an alternative energy source for brain cells, potentially counteracting the glucose hypometabolism observed in AD. Early research suggests that SGLT-2 inhibitors may reduce amyloid-beta accumulation and protect neurons from injury associated with oxidative stress [279,280,281,282]. Clinical trials to evaluate the impact of SGLT-2 inhibitors on AD progression are ongoing and showing promising insights (NCT03801642; NCT03852901) [283].

Acetylcholinesterase inhibitors (AChEIs), such as donepezil, rivastigmine, and galantamine, along with N-methyl-D-aspartate (NMDA) receptor antagonists like memantine and newer drugs like lecanemab, donanemab, and aducanumab, are used in the treatment of AD. AChEIs work by increasing the levels of acetylcholine, a neurotransmitter important for memory and learning, which is deficient in AD patients [284]. In contrast, NMDA receptor antagonists work by regulating glutamate activity, a neurotransmitter that, in excess, can contribute to neural degeneration. Memantine is typically used in moderate to severe AD [285,286]. Monoclonal antibodies like lecanemab, donanemab, and aducanumab target amyloid-beta plaques, which are believed to play a central role in AD pathology. These drugs are designed to reduce amyloid plaque buildup in the brain and are used in earlier stages of AD [287].

Additionally, brexpiprazole, an atypical antipsychotic, has been studied for its potential use in AD, particularly in managing neuropsychiatric symptoms such as agitation and aggression, which are common in patients with AD. These symptoms significantly impact the quality of life for both patients and caregivers, and current treatment options are limited. Brexpiprazole acts as a partial agonist at serotonin 5-HT1A and dopamine D2 receptors and as an antagonist at serotonin 5-HT2A and noradrenaline alpha-1B/2C receptors. This receptor profile suggests that it may help modulate neurotransmitter imbalances that contribute to neuropsychiatric symptoms in AD [288,289,290].

### 6.2. Non-Pharmacological Interventions

Currently, there is no pharmacological treatment available that can cure AD. However, emerging evidence suggests that lifestyle modifications may significantly influence the disease’s progression and symptoms. A growing body of research emphasizes the role of physical activity in preserving and enhancing brain function. Beyond the physical activity and diet mentioned above, non-pharmacological therapies are gaining attention due to their potential to alleviate the psychological and behavioral symptoms of AD, which significantly impair quality of life. A holistic study integrating music therapy, reminiscence therapy, and reality orientation therapy showed significant improvements in depression symptoms, social engagement, emotional regulation, sleep quality, and overall energy levels in patients undergoing treatment. These interventions offer a promising avenue for enhancing the quality of life in AD patients without the side effects associated with pharmacological treatments [291].

**Figure 3:**
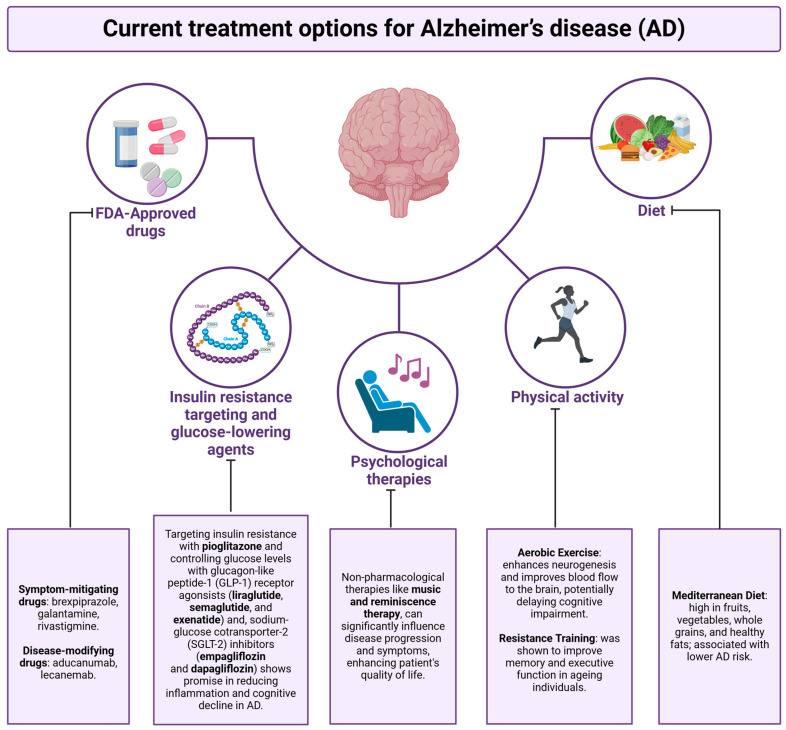
Current treatment options for Alzheimer’s disease (AD). FDA-approved drugs include both symptom-mitigating agents (brexpiprazole, galantamine, and rivastigmine) and disease-modifying treatments (aducanumab, lecanemab). Insulin resistance targeting focuses on pioglitazone, which improves insulin sensitivity via peroxisome proliferator-activated receptor gamma (PPAR-γ) activation, with the potential to reduce inflammation and cognitive decline. Growing interest has been directed toward the potential use of glucagon-like peptide-1 (GLP-1) receptor agonists in AD, based on their success in treating T2DM and their effects on neural health. GLP-1 receptor agonists, such as liraglutide, semaglutide, and exenatide exert neuroprotective actions by promoting insulin sensitivity, reducing neuroinflammation, and enhancing neuronal survival. Similarly, sodium–glucose cotransporter-2 (SGLT-2) inhibitors, another class of antidiabetic agents, are being explored for their neuroprotective potential in AD. SGLT-2 inhibitors, such as empagliflozin and dapagliflozin, work by reducing glucose reabsorption in the kidneys, thus improving glycemic control in T2DM patients and showing potential in AD treatment. Psychological therapies, such as music and reminiscence therapy, have been shown to alleviate symptoms and enhance the quality of life in patients. Physical activity, particularly aerobic exercise and resistance training, can promote neurogenesis, improve blood flow, and support cognitive functions. The Mediterranean diet, rich in fruits, vegetables, whole grains, and healthy fats, is associated with a reduced risk of AD. This figure highlights the importance of combining pharmacological and lifestyle interventions to optimize AD management and improve patient outcomes. Created with BioRender.com, accessed on 25 October 2024.

Collectively, these findings highlight the importance of a comprehensive, multifaceted approach to AD management, encompassing physical activity, diet, and non-pharmacological therapies. While pharmacological interventions remain limited, lifestyle modifications offer a viable and effective means to improve cognitive function, delay disease progression, and enhance the quality of life in individuals with AD (Figure 3).

## 7. Conclusions and Future Prospects

AD is a prevalent neurodegenerative disorder that disproportionately affects aging populations worldwide. It is primarily characterized by cognitive decline and dementia, with a multifactorial etiology influenced by genetic predispositions, advancing age, and neuroinflammatory processes. The accumulation of abnormal Aβ and tau proteins in the brain is a key factor in AD’s pathogenesis, leading to neuronal dysfunction and a reduction in brain volume. AD’s symptoms include memory retention difficulties, behavioral changes, aphasia, and spatial-temporal disorientation, significantly impairing the ability to perform daily activities. Current diagnostic approaches often involve neuroimaging techniques and biomarker assays to assess disease progression. However, no disease-modifying drug has been developed yet, though therapeutic interventions such as acetylcholinesterase inhibitors and NMDA receptor antagonists can temporarily enhance cognitive function and improve quality of life [292].

Recent research highlights a strong connection between AD and insulin resistance, similar to the relationship observed in T2DM. Individuals with T2DM are at a significantly increased risk—by 50–75%—of developing AD. Since T2DM accounts for approximately 90% of all diabetes cases worldwide, this underscores the need for routine health assessments in high-risk populations. Shared risk factors for both conditions include genetic predispositions, oxidative stress, obesity, and hypertension [293,294].

### 7.1. Pathophysiology and Emerging Insights

The link between AD and metabolic dysfunction has led to the evolving concept of AD as a metabolic disease. Insulin resistance in the brain, impaired glucose metabolism, and altered synaptic plasticity have all been implicated in AD progression. However, the exact mechanisms remain poorly understood, necessitating further investigation into how metabolic dysregulation, particularly brain-specific insulin signaling defects, contributes to neurodegeneration, Aβ accumulation, and tau pathology. Understanding how these processes interact with inflammation, oxidative stress, and neurovascular dysfunction is crucial to explaining the heterogeneity in disease progression and treatment response.

Future research should focus on identifying key regulatory pathways linking metabolic disorders and AD. This includes studying glucose transporters, insulin receptor signaling in neurons, and mitochondrial dysfunction. Such investigations may lead to novel biomarkers for early detection and new molecular targets for therapeutic intervention, especially in the context of metabolic therapies for neurodegenerative diseases.

### 7.2. Biomarkers and Diagnostic Challenges

The insidious progression of AD, often occurring silently over several years, complicates early diagnosis. Identifying early biomarkers, especially those related to insulin resistance within the brain, is critical. Recent studies have made strides in identifying potential biomarkers, such as Aβ and tau PET imaging, which may predict AD onset before clinical symptoms. Brain insulin resistance (BIR), for instance, may contribute to reduced ATP production and impaired glucose utilization, both of which are linked to cognitive deficits [295].

However, challenges persist in establishing the sensitivity and specificity of these biomarkers. For example, clusterin, a protein linked to hippocampal atrophy and cognitive decline, has shown promise, though its elevation may arise from various unrelated pathological processes. Research has demonstrated that plasma clusterin levels could serve as a prognostic marker for cognitive function. Additionally, ratios of Aβ_1-42_ to Aβ_1-40,_ along with the presence of the *ApoE4* allele, are being investigated as potential markers for predicting agitation and aggression symptoms in long-term AD patients. Yet, technical hurdles, biological variability, and high costs in clinical practice complicate biomarker validation [296,297].

### 7.3. Therapeutic Targets and Challenges

Therapeutic strategies targeting specific metabolic dysfunctions, such as insulin resistance, have shown promise. For instance, increased GSK-3β activity is a potential biomarker for AD. The hyperphosphorylation of tau protein by GSK-3β leads to the formation of NFTs, a hallmark of AD pathology. GSK-3β inhibitors, such as tideglusib and lithium carbonate, are being tested in clinical trials, but further research is required to develop effective therapies targeting this enzyme [298,299].

Additionally, diabetes medications like metformin have shown potential in reducing AD risk among long-term users, while others, such as sulfonylureas, may increase dementia risk. The relationship between obesity, T2DM, and neurodegeneration remains unclear, necessitating more research into how metabolic treatments may affect AD progression.

### 7.4. Addressing Disease Heterogeneity

AD’s heterogeneity in symptoms, pathological processes, and treatment responses poses a significant challenge in developing universally effective therapies. Variations in genetic factors, particularly the presence of the *APOE ε4* allele, complicate disease progression and treatment responses. Research utilizing multi-layered proteomic and genomic data has identified distinct AD subtypes, indicating that personalized treatment strategies may be necessary. For example, recent studies on EOAD have revealed phenotypic diversity, highlighting the need for personalized approaches [300].

Long-term cohort studies and population-based research are essential for understanding how lifestyle, diet, and physical activity influence AD development. These studies provide critical insights into disease progression and help to identify effective prevention strategies.

In summary, to address the complexity of AD and its association with metabolic dysfunctions, future research must prioritize the following:

- Detailed molecular studies to identify key regulatory pathways linking metabolic disorders to AD;
- Investigation of early biomarkers for both AD and T2DM, focusing on brain insulin resistance and glucose metabolism;
- Long-term cohort and population-based studies to assess lifestyle factors and their impact on disease progression;
- International research collaborations to standardize methodologies and enhance global understanding of AD.

While substantial progress has been made in understanding the relationship between AD and metabolic dysfunctions, significant gaps remain in our knowledge. Further research is essential to refine diagnostic tools, develop more effective treatments, and ultimately improve outcomes for individuals affected by AD.

## Figures and Tables

**Table 1:** Risk factors for Alzheimer’s disease (AD) and type II diabetes mellitus (T2DM).

Risk Factor	Description	Relevant Trial/Study
Genetic Predisposition	Specific gene variants (e.g., *APOE ε4*) increase the risk for AD, while others (e.g., *TCF7L2*) raise T2DM risk. Some genes affect both conditions through shared pathways.	Wang et al. identified 78 SNPs linked to AD, including *TP53INP1*, *TOMM40*, and *NDUFAF6*, involved in mitochondrial dysfunction and oxidative stress in both AD and T2DM (PMID: 28870582).
Oxidative Stress	Excessive ROS production damages cellular components impairs insulin signaling in T2DM and contributes to neuronal death and Aβ aggregation in AD.	Liu et al. found AD triggers oxidative stress and decreased energy metabolism in *APP*/*PS1* double-transgenic mice, mirroring metabolic dysfunction in T2DM (PMID: 31089202).
Obesity	Adipose tissue releases inflammatory cytokines, promoting insulin resistance in T2DM and neuroinflammation in AD. Obesity also strains the cardiovascular system, affecting brain health.	A 2023 observational cohort study showed brain atrophy patterns in obese individuals that resembled those in AD, suggesting obesity accelerates brain aging (PMID: 36565111).
Hypertension	Chronic high blood pressure damages blood vessels, impairing insulin delivery in T2DM and reducing cerebral blood flow in AD. It also increases the risk of vascular dementia.	A study found that middle-aged individuals with stage 1 or 2 hypertension have an 18% to 25% higher risk of developing AD later in life compared to those with normal blood pressure (PMID: 31381518).
Sedentary Lifestyle	Lack of physical activity reduces insulin sensitivity in T2DM and is associated with increased Aβ deposition and reduced brain volume in AD.	A 2022 cohort study of patients with new-onset T2DM found that regular physical activity was associated with lower risks of all-cause dementia (29%), AD (32%), and vascular dementia (41%) (PMID: 35192690).
Poor Diet	High-fat, high-sugar diets contribute to insulin resistance in T2DM and may increase inflammation and oxidative stress in the brain, promoting AD pathology.	Morris et al. demonstrated that high adherence to the MIND diet was associated with a 53% reduced rate of AD compared to those with low adherence (PMID: 25681666).
Smoking	Tobacco use increases oxidative stress and inflammation, exacerbating insulin resistance in T2DM and promoting vascular damage and Aβ accumulation in AD.	A meta-analysis of 19 studies showed that current smokers had a 30% higher risk of developing AD and a 38% higher risk of developing vascular dementia compared to non-smokers. (PMID: 17573335).
Excessive Alcohol Consumption	Heavy drinking impairs insulin sensitivity and glucose metabolism in T2DM, while in the brain, it can lead to neuroinflammation and an increased risk of cognitive decline.	A 2019 study found that in individuals with mild cognitive impairment, heavy drinking (>14 drinks/week) was associated with faster rates of cognitive decline compared to moderate drinkers (PMID: 31560382)

Abbreviations: Aβ—amyloid beta; AD—Alzheimer’s disease; *APOE ε4*—apolipoprotein E epsilon 4 allele; APP—amyloid precursor protein; MIND diet—Mediterranean-DASH diet intervention for neurodegenerative delay; NDUFAF6—NADH dehydrogenase [ubiquinone] 1 alpha subcomplex assembly factor 6; PMID—PubMed identifier; PS1—Presenilin 1; ROS—reactive oxygen species; SNP—single nucleotide polymorphism; TCF7L2—transcription factor 7-like 2; T2DM—type 2 diabetes mellitus; TOMM40—translocase of outer mitochondrial membrane 40; TP53INP1—tumor protein 53 inducible nuclear protein 1.

## Abbreviations

AD	Alzheimer’s disease
ABCA7	ATP-binding cassette sub-family A member 7
AChEIs	acetylcholinesterase inhibitors
ACTB	actin beta
ADAM10	disintegrin and metalloproteinase domain-containing protein 10
AET	aerobic exercise training
AGEs	advanced glycation end products
AKT	protein kinase B
AMPK	adenosine monophosphate-activated protein kinase
APLP1/2	amyloid precursor-like protein 1/2
APOE	apolipoprotein E
APOE-ε4	apolipoprotein E epsilon 4
APP	amyloid precursor protein
ATP	adenosine triphosphate
Aβ	amyloid-beta
BACE1	beta-site amyloid precursor protein cleaving enzyme 1
BBB	blood–brain barrier
BDNF	brain-derived neurotrophic factor
BIN1	bridging integrator 1
BIR	brain insulin resistance
BMI	body mass index
CD2AP	cortactin-CD2-associated protein
CDK5	cyclin-dependent kinase 5
CDKN1A	cyclin-dependent kinase inhibitor 1A
cFDR	conditional false discovery rate
CLU	clusterin
CNS	central nervous system
COL22A1	collagen type XXII alpha 1 chain
CR1	complement receptor type 1
CRP	C-reactive protein
CSF	cerebrospinal fluid
CT	computed tomography
CVD	cardiovascular disease
DASH	dietary approaches to stop hypertension
DEG	differentially expressed gene
dTg	double transgenic
EIF4A	eukaryotic initiation factor 4A
EOAD	early-onset AD
EPHA1	ephrin type-A receptor 1
FADH2	flavin adenine dinucleotide
FAD	familial Alzheimer’s disease
FDA	food and drug administration
GABA	γ-aminobutyric acid
GFAP	glial fibrillary acidic protein
GI	glycemic index
GLUT1/3/4	glucose transporter protein 1/3/4
GLP-1	glucagon-like peptide-1
GSK-3β	glycogen synthase kinase 3 beta
GWASs	genome-wide association studies
HbA1c	hemoglobin A1c
HIIT	high-intensity interval training
IDE	insulin-degrading enzyme
IGF-1/2	insulin-like growth factor 1/2
IL-1β	interleukin 1 beta
IL-9	interleukin 9
IRβ	insulin receptor beta
IRS-(1)	insulin receptor substrate-(1)
KD	ketogenic diet
LOAD	late-onset AD
LTP	long-term synaptic potentiation
MAPK	mitogen-activated protein kinase
MAPT	microtubule-associated protein tau
MCI	mild cognitive impairment
MCT	medium-chain triglyceride
MCP-1	monocyte chemoattractant protein-1
MeDS	Mediterranean diet
MICT	moderate-intensity continuous training
MIND	Mediterranean-DASH intervention for neurodegenerative delay
miRNAs	microRNAs
MMSE	mini-mental state examination
MRI	magnetic resonance imaging
MTBD	microtubule-binding domain
mTOR	mechanistic target of rapamycin
NADH	reduced nicotinamide adenine dinucleotide
NDUFAF6	nicotinamide adenine dinucleotide + reduced ubiquinone oxidoreductase complex assembly factor 6
NF-κB	nuclear factor kappa B
NFTs	neurofibrillary tangles
NMF	non-negative matrix factorization
NMDA	N-methyl-D-aspartate
PA	physical activity
PDK1	3-phosphoinositide-dependent protein kinase 1
PDPKs	proline-directed protein kinases
PET	positron emission tomography
PHFs	paired helical filaments
PI3K	phosphatidylinositol 3-kinase
PICALM	phosphatidylinositol binding clathrin assembly protein
PIP3	phosphatidylinositol 3,4,5-trisphosphate
PP2A	protein phosphatase 2 A
PPAR-γ	peroxisome proliferator-activated receptor gamma
PRR	proline-rich region
PSEN1	presenilin 1
PSEN2	presenilin 2
PTP1B	protein tyrosine phosphatase 1B
RAGEs	receptors for AGEs
REM	rapid eye movement
ROS	reactive oxygen species
S6K	ribosomal protein S6 kinase
sAPPα	soluble APP alpha
SCFAs	short-chain fatty acids
Ser473	serine 473
SERINC3	serine incorporator 3
SFs	straight filaments
SGLT-2	sodium–glucose cotransporter-2
SLC1A1	solute carrier family 1 member 1
SNPs	single nucleotide polymorphisms
SORL1	sortilin-related receptor 1
T2DM	type 2 diabetes mellitus
Thr308	threonine 308
TMA	trimethylamine
TMAO	trimethylamine N-oxide
TNF-α	tumor necrosis factor-alpha
TOMM40	translocase of outer mitochondrial membrane 40
TP53INP1	tumor protein p53 inducible nuclear protein 1
TZDs	Thiazolidinediones
UDP-GlcNAc	uridine diphosphate N-acetylglucosamine
VIM	Vimentin
WHO	world health organization
YOAD	young-onset AD
ZMIZ1	zinc finger MIZ-type containing 1
β-CTF	β-carboxy-terminal fragment
